# New immunological aspects of peri-implantitis

**DOI:** 10.31744/einstein_journal/2024AO0396

**Published:** 2024-02-23

**Authors:** Bárbara Bellocchio Bertoldo, Guilherme Oliveira Paulo, Taíssa Cássia de Souza Furtado, Thiago Lima Pereira, Virmondes Rodrigues, Denise Bertulucci Rocha Rodrigues, Juliana Barbosa de Faria, Rodrigo César Rosa, Sanívia Aparecida de Lima Pereira

**Affiliations:** 1 Universidade Federal do Triângulo Mineiro Uberaba MG Brazil Universidade Federal do Triângulo Mineiro, Uberaba, MG, Brazil.; 2 Centro Universitário Funorte Uberaba MG Brazil Centro Universitário Funorte, Uberaba, MG, Brazil.; 3 Universidade de Uberaba Uberaba MG Brazil Universidade de Uberaba, Uberaba, MG, Brazil.

**Keywords:** Hypoxia-inducible factor 1, Interleukin-10, Peri-implantitis, Tumor necrosis factor-alpha, Vascular endothelial growth factors

## Abstract

**Objective:**

This study aimed to compare the levels of HIF1-α, VEGF, TNF-α, and IL-10 in the peri-implant crevicular fluid of patients with and without peri-implantitis.

**Methods:**

Forty patients, comprising 16 with and 24 without peri-implantitis were selected.

**Results:**

Patients with peri-implantitis exhibited significantly higher HIF-1α levels than those without peri-implantitis (p=0.0005). TNF-α revealed significant positive correlations with IL-10 (p=0.0008) and VEGF (p=0.0246), whereas HIF-1α and IL-10 levels (p=0.0041) demonstrated a negative and significative correlation in the peri-implantitis group.

**Conclusion:**

This study, for the first time demonstrates the balance of HIF-1α, TNFα, IL-10, and VEGF in peri-implantitis. It shows an elevated HIF-1α levels in patients with peri-implantitis, which could have stemmed from persistent inflammation- triggered hypoxia. Furthermore, the positive correlation between TNF-α and VEGF suggests intensified proinflammatory activity in peri-implantitis. Nevertheless, further studies are essential to understand these immune dynamics in peri-implantitis.

## In Brief

The authors compared the levels of HIF1-α, VEGF, TNF-α, and IL-10 in peri-implant crevicular fluid between patients with or without peri-implantitis. HIF-1α levels were significantly high in the peri-implantitis possibly due to hypoxia triggered by persistent inflammation.

## Highlights

▪ Higher levels of HIF-1α in patients with peri-implantitis occurred possibly due to persistent hypoxia triggered by inflammation.
▪ Tissue hypoxia in peri-implantitis induced increase in HIF-1α consequently increased VEGF and angiogenesis, contributing to the persistence of inflammation.

## Graphical abstract

**Figure:**
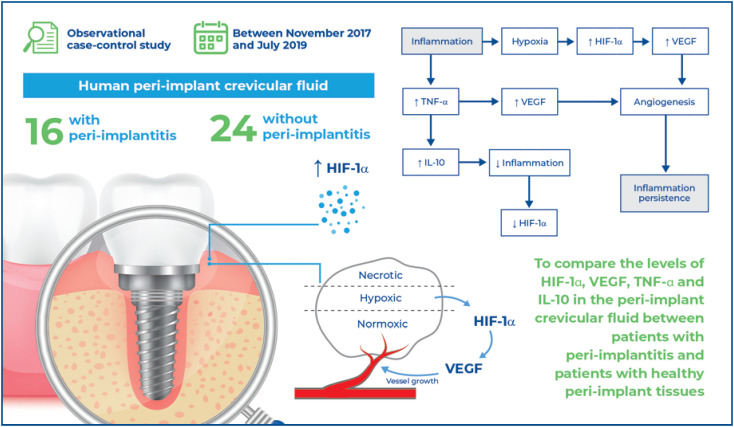

## INTRODUCTION

In recent decades, dental implants have become a popular choice for the aesthetic and functional restoration of oral edentulous spaces.^(1)^ However, several factors, including bacterial infection during or after surgery, which is considered primary etiological factor, lead to dental implant failures.^(2,3)^ Peri-implantitis is a plaque-associated pathological condition that occurs in the tissues around dental implants, characterized by inflammation in the peri-implant mucosa and subsequent progressive loss of supporting bone, jeopardizing the stability of dental implants.^(4)^

In addition to the bacterial plaques leading to peri-implant diseases, the intensity of the inflammatory response to bacterial products, such as lipopolysaccharides and endotoxins, induce the release of cytokines, leading to bone loss.^(3)^ Among these cytokines, tumor necrosis factor alpha subunit (TNF-α) and interleukin-10 (IL-10) plays opposite roles in the innate and specific immune response.^(5)^ TNF-α is a proinflammatory cytokine that induces increased adhesion and leukocyte exudation, facilitates the antimicrobial activity of neutrophils and macrophages^(6)^ and collaborates in the synthesis of other proinflammatory cytokines, being increased in both periodontitis and peri-implantitis.^(7)^ During inflammation, the increase in TNF-α is counterbalanced by the increased synthesis of the anti-inflammatory cytokine IL-10.^(6)^ IL-10 is known to have immunosuppressive properties in the control of periodontal bone loss by inhibiting the synthesis of proinflammatory cytokines, such as TNF-α.^(8)^ Furthermore, reduced levels of IL-10 have been shown to be associated with increased susceptibility to peri-implantitis.^(9)^ However, the correlation between IL-10 and TNF-α levels in peri-implantitis remains elusive.

Vascular endothelial growth factor (VEGF), a proinflammatory mediator, contributes to increased microvascular permeability and stimulates the proliferation and migration of endothelial cells, monocytes, and osteoblasts, essential for angiogenesis.^(10)^ In periodontal tissue inflammation, VEGF is associated with the progression of gingivitis to periodontitis.^(11)^ However, its role in peri-implantitis remains controversial.^(12)^

Hypoxia-inducible factors (HIFs) are heterodimeric transcription factors, comprising an oxygen-unstable alpha subunit (HIF1-α) and an oxygen-stable beta subunit (HIF1-β). HIFs play key roles in inducing cellular responses to hypoxia and act as major regulators of oxygen homeostasis in almost all cells.^(13)^ However, the expression of this transcription factor in peri-implant crevicular fluid (PICF) is yet to be elucidated.

**Figure 1:**
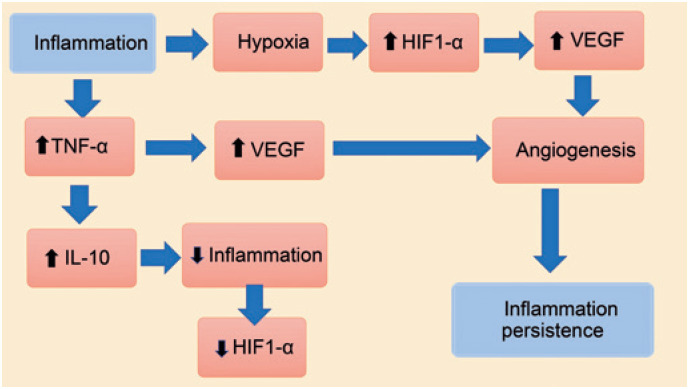
Relationship among TNF-α, HIF-1α, VEGF, and IL-10

Inflammation is known to cause tissue hypoxia,^(14)^ triggering increased synthesis HIF1-α, leading to subsequent increase in VEGF levels.^(13)^ Moreover, the increase in TNF-α also triggers an increase in VEGF levels,^(15)^ contributing to angiogenesis and persistence of inflammation.^(10,16)^ On the other hand, during inflammation, the increase in TNF-α levels is counterbalanced by the increase in the anti-inflammatory cytokine IL10,^(17)^ which triggers a reduction in HIF1-α levels^(13)^ (Figure 1).

Considering these findings, we hypothesized peri-implantitis increases the expression of VEGF, HIF-1α, TNF-α, and IL-10 in the PICF. Given that the analysis of PICF is a non-invasive method allowing the investigation of a variety of inflammatory markers, and that there are still many gaps in the understanding of the pathogenesis of peri-implantitis, the present study is justified.

## OBJECTIVE

To compare the levels of HIF-1α, VEGF, TNF-α, and IL-10 in the peri-implant crevicular fluid between patients with or without peri-implantitis.

## METHODS

This observational case–control study was conducted between November 2017 and July 2019 at a private dental clinic in the city of Uberaba, Minas Gerais, Brazil.

### Patients: eligibility criteria

In this study, the implants from 40 patients with a clinical and radiographic diagnosis of peri-implantitis (PP; case; n=16) and with healthy peri-implant tissues (HP; control; n=24), were collected. Implants with the greatest probing depth were selected when a patient had more than one implant with peri-implantitis. In the PP Group, all patients with peri-implantitis treated between November 2017 and July 2019 with peri-implant support therapy were included. For the HP Group selection, demographic data such as age, sex, and ethnicity were evaluated for a homogeneous distribution between the groups.

Medical and dental information was obtained from patients who agreed to participate in the study and met the inclusion and exclusion criteria. The inclusion criteria for patient selection in PP Group included the presence of peri-implant signs of inflammation, radiographic evidence of bone loss following initial healing, and radiographic bone level ≥3mm in combination with bleeding of probing and probing depths ≥6mm.^(18)^ Patients without erythema, bleeding on probing, swelling, suppuration and no increase in probing depth compared to previous exams were included in the HP Group (control).^(4)^ All patients were in good general health, have no parafunctional habits, and signed an informed consent form. The implants were inserted for at least six months. Patients who underwent periodontal or peri-implant anterior therapy, showed the presence of relevant and uncompensated systemic diseases, such as diabetes and osteoporosis, used antibiotics or anti-inflammatory medication in the last six months, had smoking or chronic alcoholism, were pregnant, under 18 years of age, had implants with mobility or suppuration, did not agree to sign the consent form were excluded from the study. All eligible patients were informed about the nature of the study and the potential risks and benefits of participating by signing an informed consent form. All the clinical investigations were conducted in accordance with the principles of the Declaration of Helsinki.^(19)^

### Clinical evaluation

The following parameters were evaluated at six sites of each implant (mesiobuccal, bucal, distobuccal, mesiolingual, lingual, and distolingual)^(20)^ using a periodontal probe PCPUNC-15BR (Hu-Friedy, São Paulo, Brazil). Marginal bleeding: the presence or absence of bleeding was recorded by passage of the periodontal probe along the soft tissue margin; suppuration: presence or absence of spontaneous suppuration or probing; depth of probing: distance (mm) between the margin of the mucosa to the bottom of the sulcus or peri-implant pocket.^(19)^

### Collection of peri-implant crevicular fluid

For the collection of PICF, the selected implants were isolated using a sterile gauze, and the collection sites were gently dried with an air syringe. Four cones of absorbent paper number 40 were placed individually at each collection site (mesiobuccal, buccal, distobuccal, mesiolingual, lingual^(19)^ and distolingual), approximately 2mm into the sulcus/pocket for 30 seconds each. Cones contaminated with blood or saliva were excluded. Subsequently, the cones were placed in an Eppendorf® tube containing 0.5mL phosphate-buffered saline (PBS) solution (pH 7.2) containing 1.9g potassium phosphate monobasic (KH_2_PO_4_), 5.1g sodium phosphate (Na_2_HPO_4_), 42.5g sodium chloride (NaCl), and 500mL ultra-purified water, distilled in Milli QÒ apparatus (Millipore). The samples were frozen at 70°C.

### Quantification of HIF-1*α*, VEGF, TNF-*α* and IL-10 in the PICF

The PICF samples were centrifuged at 5000 × *g* for 15 min at 4°C. Aliquots of each sample were analyzed by enzyme-linked immunosorbent assay (ELISA) to determine the levels of HIF-1α, VEGF, TNF-α and IL-10 using the specific antibody pairs. The following kits, Human/Mouse Total HIF-1 alpha/HIF1A DuoSet IC ELISA; catalog number: DYC1935-5, Human VEGF DuoSet ELISA; Catalog number: DY293B, Human IL-10 DuoSet ELISA; Catalog number: DY217B, Human TNF-alpha DuoSet ELISA; Catalog number: DY210, were used for ELISA according to the manufacturer’s recommendations (Quantikine, R&D Systems, Minneapolis, MN, USA). Briefly, high-sensitivity flat bottom 96-well plates (NUNC-Maxisorp, Rochester, NY, USA) were sensitized with 50μL of primary monoclonal antibody in bicarbonate carbonate buffer (pH = 9.4) for 18 hours at 4°C. Subsequently, they were washed with PBS/0.05% Tween in an automatic washer, blocked with 200μL PBS/2% bovine serum albumin (BSA) for 4 hours at room temperature, and washed again with PBS/0.05% Tween. Samples of tissue supernatants diluted 1:1 in 1% PBS/BSA were added to the plates, and in parallel, a standard curve was run with serial dilutions of the respective recombinant cytokines. Samples along the curve were incubated for 18 hours at 4°C. The wells were then washed with PBS/Tween, dispensed with the respective biotin-conjugated secondary monoclonal antibody at 100μL/well, and incubated for 2 hours at room temperature. After washing with PBS/Tween solution, streptavidin-conjugated alkaline phosphatase (100μL/well) in PBS/1% BSA was added, and the solution was stood at room temperature for 2 hours. Afterward, the plates were washed with PBS/Tween and the substrate inducer of enzymatic activity p-nitrophenyl phosphate at 100μL/well was added. The absorbance was measured at 450nm using an automated ELISA reader (Enspire, Perkin Elmer, USA). The concentrations of the mediators were determined by linear regression of the absorbance values obtained from the curves and expressed in pg/mL. The results were calculated using standard curves created for each assay. The ELISA was performed in a blinded manner.^(19)^

The study was approved by the Ethics Committee on Human Research of the *Universidade de Uberaba*/MG, Brazil (CAAE: 64947717.0.0000.5145; # 2.457.394).

### Statistical analyses

In the present study, the sample size was calculated considering a significance level of 5% and a statistical power of at least 85%, thus minimizing the chances of type I and type II errors.

Statistical analyses were performed using Excel 2007 for Windows (Microsoft, USA) and StatView (Abcam, USA). The Kolmogorov–Smirnov test was used to verify the normal distribution of quantitative variables. Continuous variables that presented normal distribution were expressed as mean ± standard deviation and those with non-normal distribution were expressed in median and percentiles. The variables with a normal distribution and homogeneous variance were analyzed using Student’s *t*-tests for comparison of the two groups. Variables that did not present a normal distribution or did not have homogeneous variance were analyzed using the Mann-Whitney tests. Ethnicity and sex between groups were compared using Fisher’s exact test, wherein Spearman’s correlation test was performed to analyze the correlations. Differences with a p<0.05 were considered significant.

## RESULTS

The demographic characteristics and peri-implant clinical data of patients in the PP (n=16) and HP (n=24) Groups are shown in table 1. The groups showed a homogeneous distribution but did not show significant differences in ethnicity, sex, or age.

**Table 1:** Demographic characteristics and peri-implant clinical data from the patients with or without peri-implantitis

	PP Group (n=16)	HP Group (n=24)
Ethnicity† (C:NC)	14:2	21:3
Gender‡ (M:F)	6:10	9:15
Age§ (years; mean ± SD)	56.06±3.711	52.17±2.144
MB#	16	0
PD£ (mm; mean ± SD)	5.719±0.2540*	2.354±0.2321*

† Fisher exact test, p=1

‡ Fisher exact test, p=1

§ Student’s *t*-test, p=0.788

# Student’s *t*-test, p<0.0001;

£ Mann-Whitney test, p<0.0001

* indicates statistically significant differences.

SD: standard deviation; C: caucasian; NC: non-caucasian; M: male; F: female; MB: marginal bleeding; PD: probing depth; PP: patients with peri-implantitis; HP: patients without peri-implantitis.

No marginal bleeding or bone loss was observed upon radiographic examination in the HP Group. In the PP Group, marginal bleeding was observed at all sites p<0.0001; Table 1). No suppuration or mobility was observed for any implant in either group. Other clinical data of the HP and PP Groups are presented in table 2.

**Table 2:** Clinical data of the patients with or without peri-implantitis

Perimplant clinical characteristics		PP Group n=16 (100%)	HP Group n=24 (100%)
Total number of implants (mean±SD)		4.31±2.02	3.7±1.87
Implant placement region†	Posterior	11 (68.75)	18 (75)
	Anterior	5 (31.25)	6 (25)
History of periodontitis*‡	Present	11 (68.75)	0
	Absent	5 (31.25)	24 (100)
Type of edentulism§	Total	10 (62.5)	10 (41.66)
	Partial	6 (37.5)	14 (58.33)
Implant loading period (Months) (mean±SD)^#^		10.56±2.42	11.23±1.88

† Fisher’s exact test, p=0.727

‡ Fisher’s exact test, p<0.0001

§ Fisher’s exact test, p=0.337

* indicate statistically significant differences

SD: standard deviation; PP: patients with peri-implantitis; HP: patients without peri-implantitis.

**Figure 2:**
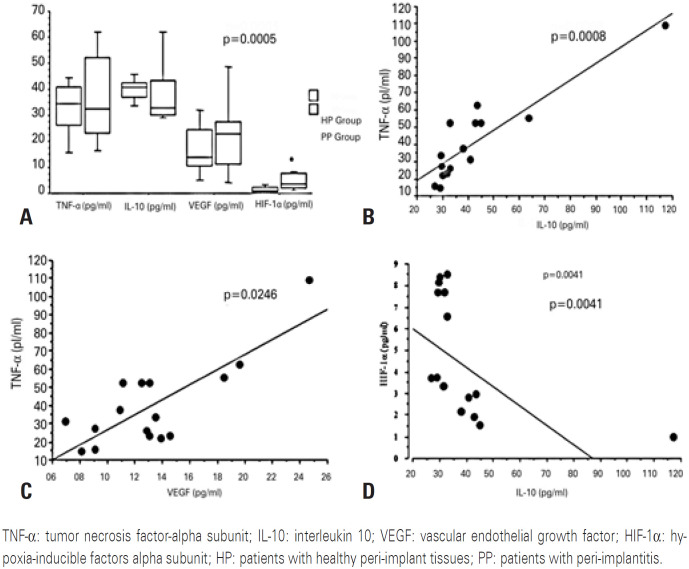
Expression of TNF-*α*, IL-10, VEGF, and HIF-1α in the peri-implant crevicular fluid of patients with or without peri-implantitis. (A) Comparison between the TNF-α, IL-10, VEGF, and HIF-1α levels in PP and HP Groups; (B) Correlation between levels of TNF-α and IL-10 in PP Group; (C) Correlation between TNF-α and VEGF levels in the PP Group; (D) Correlation between HIF-1α and IL-10 in the PP Group

The PP Group had significantly higher levels of HIF-1α than the HP Group (p=0.0005; Figure 2A). No significant difference was observed in TNF-α, IL-10, and VEGF levels between the groups. Nevertheless, the expression of TNF-α and IL-10 and that of TNF-α and VEGF showed significant positive correlations (p=0.0008 and 0.0246, respectively); Figure 2B and C) in the PP Group. In contrast, the levels of HIF-1α and IL-10 were significantly negatively correlated in the PP Group (p=0.0041; Figure 2D).

In the present study, most of the implants analyzed were from the posterior region, with 11 (68.75%) cases in the PP Group and 18 (75%) in the HP Group. The history of periodontitis between the two groups differed significantly (68.75% *versus* 0% in PP *versus* HP; p<0.0001). In the PP Group, 10 (62.5%) patients had total edentulism and 6 (37.5%) had partial edentulism. In the HP Group, 41.66% (n = 10/24) had total edentulism, while 58.33% (n=14/24) had partial edentulism. However, the implant loading period between the PP (10.56±2.42 months) and HP (11.23±1.88 months) groups did not differ significantly (Table 2).

## DISCUSSION

Peri-implantitis, despite its substantial impact on dental implants, remains a condition with ambiguous definitions and diverse etiologies, challenging our comprehension of its immunoinflammatory dynamics.^(3,9,21)^ In this study, elevated levels of HIF1-α were observed in patients with peri-implantitis, unveiling a potential association with persistent inflammation-triggered hypoxia. In addition, increased TNF*α* levels concomitantly increased the levels of IL-10 and VEGF. On the other hand, increased levels of HIF-1α were associated with decreased levels of IL-10. To our knowledge, our study is the first to scrutinize the intricate balance between these cytokines in peri-implantitis.

HIF-1α plays a key role in inducing cellular responses to hypoxia and functions as a major regulator of oxygen homeostasis in various cells.^(13)^ Therefore, hypoxia emerges as an important factor in the pathogenesis of peri-implant diseases, paralleling observations in gingival biopsies of periodontal tissues.^(14)^ Nevertheless, increased HIF-1α levels may induce angiogenesis at the site, favoring a greater influx of blood, which consequently may cause edema, flushing and bleeding on probing, as seen in the peri-implant mucosa of patients with peri-implantitis. However, despite the simplicity and non-invasiveness of collection of PICF, no prior study has evaluated this transcription factor in the PICF of individuals with peri-implantitis. In this study, we demonstrated significantly elevated HIF-1α levels in the PP Group compared to the HP Group. Furthermore, our findings revealed no significant difference between the levels of TNF-α, IL-10, and VEGF between the PP and HP Groups, which could be due to the small number of patients with peri-implantitis. However, we observed a significantly positive correlation between TNF-α and IL-10 levels in the PP Group. Although some isolated studies of TNF-α and IL-10 have already been performed in peri-implantitis,^(22)^ the correlation between these cytokines and peri-implant disease was unknown. TNF-α and IL-10 are produced by activated macrophages and play opposite roles in the innate and adaptive immune responses.^(23)^ TNF-α is a proinflammatory cytokine that causes increased adhesion and leukocyte migration to the tissue space, facilitating the antimicrobial activity of neutrophils and macrophages.^(24)^ An increase in TNF-α levels induces increased production of anti-inflammatory cytokine IL-10.^(17)^ Taken together, the concomitant increase in TNF-α and IL-10 levels could explained as a result of the ability of TNF-α to stimulate IL-10 production in an attempt to reduce inflammation and associated tissue damage.

VEGF, a proinflammatory mediator plays a crucial role in angiogenesis.^(10)^ VEGF contributes to the severity of inflammation in gingivitis and periodontitis by inducing the emergence of new blood vessels, which facilitate the transport of proinflammatory cells, nutrients, and oxygen to inflamed tissues.^(16)^ Previous studies on peri-implantitis have demonstrated controversial levels of VEGF in peri-implant tissues;^(12)^ however, in this study, no significant difference was observed in the levels of this growth factor between groups. Rather, the findings revealed a significant positive correlation between TNF-α and VEGF in the peri-implantitis group, consistent with those of a previous study demonstrating that increased TNF-α induces the expression of VEGF.^(15)^ Together, these findings suggest that a concomitant increase in TNF-α and VEGF, which have proinflammatory activity, contributes to the intensification of the inflammation in the peri-implantitis. This was the first study to correlate TNF-α and VEGF in the peri-implantitis.

The anti-inflammatory cytokine IL-10 produced by T and B lymphocytes, activated monocytes, and macrophages has immunosuppressive properties in the control of periodontal bone loss.^(8)^ A recent study in mice with arthritis has shown that high concentrations of HIF-1α are associated with increased IL-10 production by B cells, thus contributing to the reduction of the inflammatory response in the joints.^(25)^ However, in the present study, a decrease in HIF-1α was found to be associated with an increased IL-10 synthesis. Our findings corroborate a study in mice infected with *Histoplasma capsulatum*, which demonstrated that the absence of HIF-1α in the infected mice caused an increase in the IL-10 cytokine and consequent increase in the fungal load and death of most animals.^(26)^ Together, these findings suggest that the increase in the anti-inflammatory cytokine IL-10 could reduce the inflammatory condition in peri-implantitis with an improvement of tissue hypoxia, evident from the reduced HIF-1α levels. This reduction in inflammation can minimize tissue damage, although it can also contribute to the survival of pathogenic bacteria in peri-implant tissues. However, the relationship between the production of HIF-1α and IL-10 is complex and seems to depend on the cell type and the inflammatory environment in which it occurs,^(25)^ and necessitates further exploration, especially in the unique context of peri-implantitis.

Furthermore, in the present study, the history of periodontitis differed significantly between the two groups. This finding corroborates systematic reviews demonstrating that subjects with a history of periodontitis may be at a greater risk of peri-implant infections.^(27,28)^

Although the present study was the first to assess the TNF-α, IL-10, VEGF, and HIF1-α immune balance in peri-implantitis, it has limitations since we evaluated the local immune response of the peri-implant condition at a given time. As there was no prospective assessment, the present study only assessed the peri-implant condition at the time of PICF collection and not during the disease.

## CONCLUSION

The study’s findings revealed that individuals with peri-implantitis exhibited significantly elevated levels of HIF-1*α* compared to the control group, implying a substantial role for hypoxia in the pathogenesis of peri-implant disease. Furthermore, the simultaneous increase in TNF-α and VEGF was identified as a potential contributor to the heightened inflammatory response in peri-implantitis, a response that appeared to be counterbalanced by the elevated levels of IL-10. The augmentation of IL-10, an anti-inflammatory cytokine, demonstrated a capacity to ameliorate inflammation, thereby leading to a subsequent reduction in tissue hypoxia. In summary, our findings suggest a dynamic interplay in peri-implantitis, where the concomitant rise in TNF-α and VEGF accentuates proinflammatory activity. Paradoxically, the proinflammatory cytokine TNF-α appears to stimulate the synthesis of the anti-inflammatory cytokine IL-10, potentially contributing to inflammation reduction and the consequential alleviation of local hypoxia, as evidenced by the reduction of HIF1-α. However, it is crucial to acknowledge that this study represents the first attempt to correlate TNF-α, IL-10, VEGF, and HIF1-α in peri-implantitis. Consequently, further studies are essential to provide a more comprehensive understanding of the intricate immune balance in this condition.
